# Work-Family Conflict and Mindful Parenting: The Mediating Role of Parental Psychopathology Symptoms and Parenting Stress in a Sample of Portuguese Employed Parents

**DOI:** 10.3389/fpsyg.2019.00635

**Published:** 2019-03-22

**Authors:** Helena Moreira, Ana Fonseca, Brígida Caiado, Maria Cristina Canavarro

**Affiliations:** Center for Research in Neuropsychology and Cognitive Behavioral Intervention, University of Coimbra, Coimbra, Portugal

**Keywords:** mindful parenting, parents, parenting stress, parental anxiety symptoms, parental depressive symptoms, work-family conflict

## Abstract

**Aims:** The aims of the current study are to examine whether parents’ work-family conflict, emotional distress (anxiety/depressive symptoms and parenting stress) and mindful parenting vary according to the type of employment (full-time, part-time, and occasional), the type of work schedule (fixed, flexible, and shift), and the number of working hours per week and to explore whether parental emotional distress mediates the association between work-family conflict and mindful parenting dimensions.

**Methods:** A sample of 335 employed parents (86.3% mothers) of children and adolescents between the ages of 1 and 19 years old completed a sociodemographic form and measures of work-family conflict, anxiety/depression symptoms, parenting stress, and mindful parenting. The differences in study variables among types of employment, work schedules and number of weekly working hours were analyzed. A path model was tested through structural equation modeling in AMOS to explore the indirect effect of work-family conflict on mindful parenting dimensions through anxiety, depression and parenting stress. The invariance of the path model across children’s age groups (toddlers, preschool and grade school children, and adolescents) and parents’ gender was also examined.

**Results:** Parents with a shift work schedule, working full-time and 40 h or more per week, presented significantly higher levels of work-family conflict than those with a fixed or flexible schedule, working part-time and less than 40 h per week, respectively. Parents with a flexible work schedule presented significantly higher levels of self-regulation in parenting and of non-judgmental acceptance of parental functioning than parents with a shift work schedule. Higher levels of work-family conflict were associated with lower levels of mindful parenting dimensions through higher levels of anxiety/depression symptoms and parenting stress. The model was invariant across children’s age groups and parents’ gender.

**Discussion:** Work-family conflict is associated with poorer parental mental health and with less mindful parenting. Workplaces should implement family-friendly policies (e.g., flexible work arrangements) that help parents successfully balance the competing responsibilities and demands of their work and family roles. These policies could have a critical impact on the mental health of parents and, consequently, on their parental practices.

## Introduction

Balancing the dual demands of work and parenting pose a great challenge to contemporary parents. Labor markets are becoming increasingly competitive and insecure, requiring parents to make a great commitment to work and dedicate much of their family and personal time to their jobs. In Portugal, the employment rate for all Portuguese mothers (aged 15–64) with at least one child aged 0–14 is 75.7% (68.4% working full-time), which is higher than the OECD average (66.2%) and one of the highest rates among OECD countries. The proportion of children (aged 0–14) that live in households where both parents work full-time is 61.9%, which is also higher than the OECD average of 56%. In addition, family-friendly workplace policies, which are key determinants of a family ability to reconcile work and family life, are not employed by the majority of companies in Portugal. For instance, only 34.5% of companies report providing flexible working time arrangements, such as the possibility to accumulate hours for days off (full or half days) and to vary the start and end of daily work, to at least some employees (the OECD average is 53.4%) (OECD, 2019).

Some studies show that the pressure parents feel on their jobs (e.g., to display greater commitment and dedication) along with certain working conditions (e.g., inflexible work schedules and work overload) may create a work-family conflict and have a strong impact on parents’ well-being and on their parental behaviors (e.g., Costigan et al., 2003). When experiencing this conflict between the incompatible demands of work and family, parents may feel high levels of distress (e.g., anxiety, depression, and parenting stress), which, in turn, can have a negative impact on their parenting behaviors and practices, including their ability to adopt a mindful approach to parenting.

### Mindful Parenting

Over the last decade, several researchers, particularly those interested in family and parent–child relationships, have been showing an increasing interest in the study of the applications of mindfulness to the parenting context (e.g., Dumas, 2005; Singh et al., 2007; Bögels and Restifo, 2014; Parent et al., 2016a). Mindful parenting is a parental approach that can be simply described as bringing non-judgmental, compassionate, and present-centered awareness into parent–child interactions (Kabat-Zinn and Kabat-Zinn, 1997; Bögels and Restifo, 2014; Bögels et al., 2014). According to Kabat-Zinn and Kabat-Zinn (1997), the authors who made popular the term *mindful parenting*, this parental approach “means seeing if we can remember to bring this kind of attention and openness and wisdom to all moments with our children” (p. 25); it “is a continual process of deepening and refining our awareness and our ability to be present and to act wisely” (p. 28).

Mindful parenting offers an alternative to the automatic pilot mode in which so many parents live (Kabat-Zinn and Kabat-Zinn, 1997), allowing them to *be* in the present moment when interacting with their children, to be sensible and responsive to their child’s needs, and to exert self-regulation in difficult moments with the child and thereby choose parenting behaviors (rather than reacting automatically) that are in accordance with their parental values and goals (Duncan et al., 2009). Being a mindful parent implies that parents are able and willing to be open and receptive to their children’s behaviors, thoughts and emotions, without judging them and automatically reacting to them, so parents can truly see and understand their children and act in a sensitive manner (Bluth and Wahler, 2011).

For Duncan et al. (2009), mindful parenting encompasses several dimensions, such as the ability to listen to the child with full attention (i.e., being fully present and with complete attention to the child in parent–child interactions), greater self-regulation in parenting (i.e., being able to regulate the emotions and behaviors in parent–child interactions), greater emotional awareness of the child (i.e., being able to notice and correctly identify the child’s emotions), an attitude of compassion toward the child (i.e., being able to be kind, sensible and responsive to the child’s needs), and a non-judgmental acceptance of parental functioning (i.e., being able to accept without criticizing the self as a parent) (Duncan et al., 2009; de Bruin et al., 2014; Moreira and Canavarro, 2017).

Bringing mindful awareness to parent–child interactions promotes a higher quality parent–child relationship (Duncan et al., 2009). There is some evidence that mindful parenting is associated with less parenting stress (Beer et al., 2013; Bögels and Restifo, 2014; Bögels et al., 2014; Gouveia et al., 2016; Moreira and Canavarro, 2018b), more positive parenting styles and practices (Williams and Wahler, 2010; de Bruin et al., 2014; Gouveia et al., 2016; Parent et al., 2016b; Moreira and Canavarro, 2017), and a more secure attachment relationship between the parents and the child (Medeiros et al., 2016; Moreira et al., 2018b). Mindful parenting was also shown to be associated with several positive outcomes for children and adolescents, such as lower levels of internalizing and externalizing problems (Geurtzen et al., 2015; Parent et al., 2016b), greater psychosocial wellbeing (Medeiros et al., 2016), and a lower likelihood of substance use (Turpyn and Chaplin, 2016). In addition, interventions aimed at promoting the development of mindful parenting skills (e.g., Bögels and Restifo, 2014) proved to be highly effective in reducing parenting stress and promoting positive parenting practices and the psychological adjustment of parents and children in different groups of parents (e.g., Singh et al., 2006, 2007; van der Oord et al., 2012; Bögels et al., 2014; Potharst et al., 2017).

Mindful parenting, like other parenting styles and practices, is multiply determined and can be influenced by intersecting parent (e.g., personality, mental health, and gender), child (e.g., temperament and age), and social (e.g., parents’ work context) variables (Belsky, 1984). For instance, with regard to parent variables, we have shown that parents’ attachment and caregiving orientations (Moreira and Canavarro, 2015; Moreira et al., 2016), self-compassion and dispositional mindfulness (Gouveia et al., 2016), and self-critical rumination (Moreira and Canavarro, 2018b) were important internal variables that could play a role in mindful parenting. The gender of parents is also an important variable in determining levels of mindful parenting. In previous investigations, we have shown that women presented higher levels of mindful parenting than men, although these studies have only explored the gender differences in the total score of the mindful parenting scale and not in the different mindful parenting dimensions (Moreira and Canavarro, 2015;Medeiros et al., 2016).

Other potential determinants of mindful parenting are the parents’ mental health and their levels of parenting stress (i.e., the stress that results from perceiving the demands of parenting as exceeding personal and social resources to cope with those demands; Abidin, 1992). In fact, there is ample evidence that parental psychopathology and parenting stress are among the strongest risk factors for negative parenting behaviors (Goodman and Gotlib, 1999; Lovejoy et al., 2000; Goodman, 2007; Shea and Coyne, 2011; Goodman and Garber, 2017), which supports the hypothesis that more depressed, anxious or stressed parents would struggle more to bring mindful awareness to the relationship with their children. Although the role of parents’ mental health on mindful parenting has been little investigated, in a previous study, we found that mothers reporting clinically significant levels of anxiety and/or depression symptoms presented lower levels of all the dimensions of mindful parenting (Moreira and Canavarro, 2018a). In another study, we have also shown that higher levels of parenting stress were associated with lower levels of mindful parenting (Gouveia et al., 2016). When exploring the contribution of psychopathology and parenting stress on parenting behaviors, it is important to note that the relationship between these variables can be bidirectional. While some studies have shown that depression and/or anxiety symptoms increase the likelihood of parenting stress (Williford et al., 2007; Pritchard et al., 2012), others have demonstrated that parenting stress lead to psychopathology symptoms (Thomason et al., 2014; Weitlauf et al., 2014; Vismara et al., 2016; Rollè et al., 2017), and still others have treated parenting stress and depression symptoms as same-level variables (Ponnet et al., 2013).

Children characteristics, as their age or developmental stage, can also be important determinants of parenting. Children and adolescents of different ages pose different challenges for parents (Galinsky, 1987) and, therefore, potential differences in parenting practices and behaviors should be always considered when conducting studies that include parents of children in different stages of development. Nevertheless, previous research suggested that mindful parenting do not vary according to children’s age group. For instance, Medeiros et al. (2016) found no significant differences between parents of children aged 8–12 years and parents of adolescents aged 13–19 years and Moreira et al. (2018b) found no significant differences between parents of adolescents in the yearly and middle/late stages of adolescence. More distal variables of the social context, such as the parents’ working context, may also have an important role in determining how mindful parents can be in their parental role. However, the role of work-related variables in mindful parenting has never been investigated.

### Work-Family Conflict and Parenting

In recent decades, there has been increasing interest in understanding the influence of parents’ work context on parenting. Research on the work-family interface has shown that parents’ work experiences have a considerable impact on their parenting behaviors and on the overall quality of their family life (Crouter and Bumpus, 2001). For instance, stressful work conditions (e.g., work overload, feelings of pressure, low autonomy, long work hours, inflexible schedules or a negative work environment) have been linked to more negative (e.g., intrusive and less sensitive) parent–child interactions (Costigan et al., 2003), more harsh and less warmth and responsive parenting behaviors (Greenberger et al., 1994), lower emotional and behavior involvement in parent–child interactions (Repetti and Wood, 1997), and less frequent leisure and childcare activities with children (Bass et al., 2009; Roeters et al., 2010). Stressful working conditions have also been associated with poorer mental health in parents (Perry-Jenkins et al., 2017) and children (Johnson et al., 2013; Dockery et al., 2016). For example, some types of work schedules, such as shift work, have been associated with more mental and physical health problems (Figueiro and White, 2013; Cho, 2018).

Reconciling work and family-related responsibilities, often without any support from family or others and frequently in competitive, stressful, and insecure jobs, is a challenge that many working parents face today. This conflict between the competing responsibilities and demands of work and family contexts has been labeled a “work-family conflict” and was defined as “a form of inter-role conflict in which the role pressures from the work and family domains are mutually incompatible so that participation in one role is made more difficult by participation in another role” (Greenhaus and Beutell, 1985, p. 77). This interrole conflict may have two distinct directions – work interfering with family and family interfering with work – each presenting distinctive determinants and consequences (Byron, 2005; Michel et al., 2011). In this study, the term *work-family conflict* is used to describe the interference of work with family.

The work-family conflict has been linked to particular work conditions, including working long hours and having inflexible working schedules (Cooklin et al., 2015a), having a shift work schedule (Barnett et al., 2008; Mauno et al., 2015), and having a full-time job as opposed to a part-time job (Higgins et al., 2000; Hill et al., 2004). Previous research has also shown that work-family conflict may depend on the parents’ gender. With the increase in the number of dual-earner families over time and the fact that parents have more traditional implicit gender-role stereotypes (i.e., women’s role as homemaker and men’s role as economic provider) than non-parents (Endendijk et al., 2018), the work-family conflict has been an issue for both parents. However, mixed results with regard to gender differences have been reported in the literature. While most studies show higher levels of conflict among women (Cinamon and Rich, 2002; Lee et al., 2003; Ahmad and Omar, 2008), others have found higher levels of conflict among men (Allen and Finkelstein, 2014), and still others have found no differences between men and women (Duxbury and Higgins, 1991).

Work-family conflict is currently considered a major social determinant of parents’ family environment and parenting behaviors (Dinh et al., 2017). Several studies have shown that parents experiencing higher levels of work-family conflict have lower quality parent–child interactions (Lau, 2010; Vieira et al., 2016), which are characterized, for instance, by irritable, less warm and inconsistent parenting behaviors (Cooklin et al., 2015b, 2016). Parents experiencing this interrole conflict were also shown to report lower parental self-efficacy (Cinamon et al., 2007) and lower parental satisfaction (Vieira et al., 2012). Work-family conflict has also been linked to child mental health. For instance, some studies found that work-family conflict was positively associated with children’s emotional distress (Strazdins et al., 2013; Vieira et al., 2016; Vahedi et al., 2018) and negatively associated with children’s self-esteem (Lau, 2010).

One of the possible vehicles through which work-family conflict can impact parenting behaviors, including mindful parenting behaviors, is the mental health of parents and their levels of parenting stress (i.e., perceiving the actual demands of parenting as exceeding personal and social resources to cope with those demands; Abidin, 1992). In fact, several studies have shown that higher levels of work-family conflict are associated with poorer parents’ mental health (Kinnunen et al., 2004; Cooklin et al., 2015a; Westrupp et al., 2016) and with higher levels of parenting stress (Kinnunen et al., 2004; Vieira et al., 2012).

### The Present Study

This study had two main goals. First, we aimed to explore whether work-family conflict, anxiety/depression symptoms, parenting stress, and mindful parenting could vary according to parents’ gender and to parents’ key working characteristics, including type of work schedule (fixed, flexible, or shift work), type of employment (full-time, part-time/occasional), and number of weekly working hours (less than 40 h, 40 h or more). Based on previous investigations, we expected to find lower levels of work-family conflict, anxiety/depression symptoms and parenting stress, and higher levels of mindful parenting among women compared to men, and among parents with a flexible work schedule, parents with a part-time job and parents who work fewer hours per week.

The second goal of this study was to investigate whether work-family conflict could play a role in parents’ ability to be mindful in the relationship with their children and whether this relationship could be mediated by parenting stress and by anxiety and depressive symptoms. To understand whether this model could be applicable to mothers and fathers and to various developmental stages of the child, we included mothers and fathers of toddlers (1–3 years), preschool and grade school children (4–11 years) and adolescents (12–19 years) in our sample, and we tested the invariance of the model with respect to the parents’ gender and the children’s age group. Based on previous studies showing that work-family conflict is associated with negative parenting experiences (e.g., Dinh et al., 2017), we expected that higher levels of work-family conflict could be directly associated with lower levels of mindful parenting. In addition, based on studies that demonstrated that work-family conflict is a risk factor for poor parental mental health (e.g., Cooklin et al., 2015a) and for higher levels of parenting stress (e.g., Vieira et al., 2012) and that parent mental health and parenting stress are associated with more negative parenting behaviors (e.g., Shea and Coyne, 2011), including lower levels of mindful parenting (Moreira and Canavarro, 2018b), we hypothesized that parents’ anxiety and depression symptoms and parenting stress would mediate the relationship between work-family conflict and mindful parenting.

## Materials and Methods

### Participants

As presented in Table 1, the sample comprised 335 parents (86.3% mothers) of children and adolescents aged 1–19 years. As the majority of parents had more than one child, they were asked to choose one of their children when completing the mindful parenting questionnaire. Table 1 presents the sociodemographic characteristics of the child on whom parents focused when answering the questionnaire.

**Table 1:** Sociodemographic and work-related characteristics.

	*N* = 335
**Parents’ characteristics**	
Parents’ gender *n* (%)	
Female	289 (86.3%)
Male	46 (13.7%)
Age (years) *M* (*SD*); range	38.86 (5.59); 20–52
Parents’ education *n* (%)	
Basic or secondary studies	115 (34.3%)
Higher education (bachelor’s, master’s or doctoral degree)	220 (65.7%)
Area of residence *n* (%)	
Rural	142 (42.4%)
Urban	193 (57.6%)
Parents’ cohabitating status *n* (%)	
Living with a partner	289 (86.3%)
Not living with a partner	45 (13.4%)
Number of children	
One	139 (41.5%)
More than one	196 (58.5%)
Household monthly income *n* (%)	
<2000€	216 (64.5%)
≥2000€	119 (35.5%)
Type of employment *n* (%)	
Full-time	296 (88.4%)
Part-time	34 (10.1%)
Occasional	5 (1.5%)
Work schedule *n* (%)	
Fixed	234 (69.9%)
Flexible	47 (14%)
Shift work	54 (16.1%)
Number of working hours per week *M* (*SD*); range	38.4 (8.83); 4–80
**Children’s characteristics**	
Child’s age (years) *M* (*SD*); range	7.29 (4.43); 1–19
Child’s age category *n* (%)	
Toddlers (1–3 years old)	73 (21.8%)
Preschool and grade school children (4–11 years old)	202 (60.3%)
Adolescents (12–19 years old)	60 (17.9%)
Child’s gender	
Female	157 (46.9%)
Male	178 (53.1%)

### Procedure

The sample was collected online (*n* = 266, 79.4%) and in one public basic education school in the central region of Portugal (*n* = 69, 20.6%) between December 2017 and April 2018. The only inclusion criterion was to be the parent of a child or adolescent between the ages of 1 and 19 years old. Participants who were recruited online completed the questionnaires in a data collection website (LimeSurvey^^®^^). The survey link was shared on social networks and through email. In the first page of the online survey, a brief description of the study goals, the inclusion criterion, and the ethical issues that guided the study were presented. In particular, in this first page, it was clearly stated that participation in the study was anonymous and that no identifying information could be collected. Since the data was collected online, participants did not provide written informed consent. Instead, participants provided informed consent by clicking on the option “I understood and accept the conditions of the study,” which was on the second page of the survey. Only those who selected this option were granted access to the assessment protocol. Parents who were recruited in the school received, through their children, a letter explaining the study, an informed consent form, and the questionnaires that should be completed at home and returned a week later. Research assistants collected the written informed consents and the questionnaires at the school on a date agreed upon with the class director. Authorization for the sample collection was obtained from the Ethics Committee of the Faculty of Psychology and Education Sciences of the University of Coimbra and from the board of directors of the school.

### Measures

#### Sociodemographic and Working Variables

Participants completed a sociodemographic form assessing their age, sex, cohabitating status, education, area of residence, family monthly income, number of children, and child’s sex and age. This form also asked parents about their type of employment (full-time, part-time, or occasional), number of working hours per week, and work schedule. The variable work schedule comprised three categories: a fixed work schedule (i.e., a schedule with the same number of working hours and days per week), a flexible work schedule (i.e., a schedule that allows employees to vary their workday start and finish times, choose the days they work, and/or work from home), and a shift work schedule (i.e., a work schedule in which most of the working hours fall outside a typical daytime Monday to Friday week and that can include evening shifts, night shifts, weekend work, irregular hours, on call, and split or rotating shifts).

#### Work-Family Conflict

The Portuguese version of the Work-Family Conflict (WFC) subscale of the Work-Family Conflict Scale (Haslam et al., 2015; Moreira et al., 2018a) was used to assess parents’ perceived negative impacts of work on family. This subscale has 5 items (e.g., “Working often makes me irritable or short tempered at home”) answered on a 7-point scale from 1 (*very strongly disagree*) to 7 (*very strongly agree*). The total score of this subscale is the sum of all items and higher scores indicate higher levels of conflict. Although the Work-Family Conflict Scale has also a Family-Work Conflict subscale, we have only included in the study the WFC subscale since we were only interested in assessing the interference of work with the family. The original version presented adequate internal consistency and convergent, concurrent and predictive validity. Preliminary data of the Portuguese version has also exhibited adequate internal consistency and construct validity. In the current study, the Cronbach’s alpha was 0.89.

#### Anxiety and Depression Symptoms

The Portuguese version of the Hospital Anxiety and Depression Scale (HADS; Zigmond and Snaith, 1983; Pais-Ribeiro et al., 2007) was administered to parents to assess their levels of depressive and anxious symptoms in the last seven days. The scale comprises 14 items and uses a 4-point Likert scale from 0 (*not at all/only occasionally*) to 3 (*most of the time/a great deal of the time*). The items are organized into two subscales: Anxiety and Depression. The total score of each subscale is the sum of all items, with higher scores indicating higher levels of symptomatology. The HADS is frequently used for screening anxious and depressive symptomatology in clinical settings and in the general community and it has shown robust psychometric properties in a wide range of populations and cultures. The Portuguese version has also robust psychometric properties, including adequate reliability and construct validity (Pais-Ribeiro et al., 2007). In the sample of this study, the Cronbach’s alpha coefficients were 0.84 for anxiety and 0.82 for depression.

#### Parenting Stress

To assess the distress associated with the parental role, the Portuguese version of the Parental Stress Scale (PSS; Berry and Jones, 1995; Mixão et al., 2010) was employed. The PSS has 18 items (e.g., “I feel overwhelmed by the responsibility of being a parent”) answered on a 5-point Likert scale from 1 (s*trongly disagree*) to 5 (*strongly agree*). The total score results from the sum of the items and higher scores indicate higher levels of parenting stress. The scale presented adequate psychometric properties, in the original and Portuguese versions, including adequate reliability (Cronbach’s alpha > 0.80) and construct validity in samples of parents mostly from the general community. In the current study, the Cronbach’s alpha was 0.70.

#### Mindful Parenting

Mindful parenting was assessed through the Portuguese version of the Interpersonal Mindfulness in Parenting Scale (IM-P; Duncan, 2007; Moreira and Canavarro, 2017). The Portuguese version (Moreira and Canavarro, 2017) includes 29 items that are scored on a 5-point Likert scale, from 1 (*never true*) to 5 (*always true*). The subscale scores result from the sum of the items, and higher scores suggest higher levels of each mindful parenting dimension. Parents were requested to think on only one of their children when answering the questionnaire, if they had more than one child. In the online data collection, parents were instructed to choose, preferably, their youngest child; in the data collection in schools, parents were instructed to think about the child who received the questionnaire in the school. IM-P items were distributed across the following subscales: (1) Listening with Full Attention (LFA; assesses the degree to which parents are attentive to their children and fully present in interactions parent–child interactions; e.g., “I pay close attention to my child when we are spending time together”), (2) Compassion for the Child (CC; assesses the extent to which parents are kind, sensitive and responsive to the child’s needs; e.g., “I try to be understanding and patient with my child when he/she is having a hard time”), (3) Non-Judgmental Acceptance of Parental Functioning (NJAPF; assesses an attitude of non-judgmental acceptance of the self as a parent; e.g., “When I do something as a parent that I regret, I try to give myself a break”), (4) Self-Regulation in Parenting (SR; assesses an ability to self-regulate emotions and behaviors during parent–child interactions; e.g., “In difficult situations with my child, I pause without immediately reacting”), and (5) Emotional Awareness of the Child (EAC; assesses the ability to notice and correctly identify the child’s emotions; e.g., “I can tell what my child is feeling even if he/she does not say anything”). Although the psychometric properties of the original version of IM-P scale are unknown, the scale has shown reliability and construct validity in Dutch samples (de Bruin et al., 2014). The Portuguese version (Moreira and Canavarro, 2017) have also exhibited reliability and construct validity among parents from the general community. In the current study, the Cronbach’s alphas were 0.85 (LFA), 0.63 (EAC), 0.81 (SR), 0.77 (NJPAF), and 0.83 (CC).

### Data Analyses

The Statistical Package for the Social Sciences (SPSS, version 25.0; IBM SPSS, Chicago, IL, United States) and the AMOS 20 (IBM^^®^^ SPSS^^®^^ AMOS^TM^ Version 20.0; IBM Corporation, Meadville, PA, United States) were used for data analyses. Descriptive statistics were computed for all sociodemographic and study variables. Since parents were collected through two different procedures, differences in sociodemographic and study variables between parents who participated online and parents who were collected at the school were analyzed through ANOVAs and chi-square tests. Next, controlling for the sociodemographic variables that differed significantly between parents collected online and at the school, differences in study variables were analyzed through one-way ANCOVAs (work-family conflict and parenting stress) and MANCOVAs (anxiety/depressive symptoms and mindful parenting dimensions). To examine the bivariate associations between study variables, Pearson correlations were computed. In addition, correlations between the sociodemographic/working variables and the mindful parenting dimensions were computed with the aim of identifying potential covariates that should be controlled in the path model. Correlations close to 0.10 were considered small, close to 0.30 were considered medium, and at 0.50 or higher were considered large (Cohen, 1988).

Differences in work-family conflict, anxiety/depression symptoms, parenting stress, and mindful parenting dimensions were analyzed as a function of work schedule (fixed versus flexible versus shift work), type of employment (full-time versus part-time/occasional), and number of working hours per week (less than 40 h versus 40 h or more) through ANOVAs and MANOVAs. The part-time and occasional categories were merged into one group because only five individuals (1.5%) reported having occasional work. Because the work schedule had three categories, *post hoc* comparison analyses with a Bonferroni correction were performed to ascertain which groups differed from the others.

To examine whether work-family conflict was associated with mindful parenting dimensions through parents’ anxiety and depressive symptoms and parenting stress, a path model was tested in AMOS (maximum likelihood estimation method). The model fit was considered good when χ^2^ was non-significant (*p* > 0.05), the CFI was ≥0.95, the RMSEA was ≤0.06, and the SRMR was ≤0.08 (Hu and Bentler, 1999). A bootstrap resampling procedure with 2000 samples and a 95% bias-corrected confidence interval (BC95% CI) was used to estimate the significance of the indirect effects. The specific indirect effects and their confidence intervals were estimated using an AMOS user-defined estimand. The structural invariance of the path model across children’s age groups (toddlers versus preschool and grade school children versus adolescents) and parents’ gender was tested through multigroup analyses. After examining the baseline model for each group separately, the unconstrained model (i.e., a model without equality constraints on parameters; configural invariance model) was compared with a model in which structural weights were constrained to be equal across groups. A non-significant chi-square difference (Δχ^2^) between the two models indicated that the path model was invariant across groups.

## Results

### Preliminary Analyses

Differences in the sociodemographic and working variables between parents recruited online and parents recruited in the school were analyzed. Significant differences were only found for parents’ gender, [χ^2^(1) = 6.56, *p* = 0.010], education [χ^2^(1) = 8.61, *p* = 0.003], and income [χ^2^(1) = 4.50, *p* = 0.034]. Controlling for these sociodemographic variables, differences between groups were analyzed for all the study variables. No significant differences were found for any study variable: work-family conflict [*F*(1,330) = 0.01, *p* = 0.931], parenting stress [*F*(1,330) = 0.98, *p* = 0.323], anxiety and depressive symptoms [Wilk’s lambda = 0.998, *F*(2,329) = 0.34, *p* = 0.715], and mindful parenting dimensions [Wilk’s lambda = 0.990, *F*(5,326) = 0.68, *p* = 0.643]. Therefore, the two groups were combined and analyzed together.

Correlations between study variables were also analyzed. As presented in Table 2, significant correlations were found between work-family conflict and all the remaining variables. Anxiety, depression and parenting stress were significantly and positively correlated with each other and significantly and negatively associated with mindful parenting dimensions. In addition, correlations between sociodemographic (parents’ age, gender, education, cohabitating status, income, area of residence, number of children; children’s gender and age) and working variables (work schedule, type of employment and number of working hours per week) and mindful parenting dimensions were analyzed to investigate whether any variable should be introduced as a covariate in the path model. Listening with full attention was significantly correlated with parents’ gender (0 = men, 1 = women; *r* = -0.12, *p* = 0.034) and child’s gender (0 = boy, 1 = girl; *r* = -0.13, *p* = 0.018); self-regulation was significantly correlated with work schedule (0 = fixed/shift work, 1 = flexible; *r* = 0.12, *p* = 0.034) and child’s gender (*r* = -0.14, *p* = 0.008); non-judgmental acceptance was significantly correlated with education (0 = basic/secondary education, 1 = higher education; *r* = 0.15, *p* = 0.006), income (0 = 2000€, 1 = ≥2000€; *r* = 0.12, *p* = 0.023), and work schedule (*r* = 0.13, *p* = 0.016); and compassion for the child was significantly correlated with child’s gender (*r* = -0.14, *p* = 0.009).

**Table 2:** Correlations among study variables.

Study variables	*M (SD);* range	1	2	3	4	5	6	7	8
(1) Work-family conflict	19.24 (7.47); 5–35	–							
(2) Anxiety symptoms	6.68 (3.82); 0–19	0.26^∗∗^	–						
(3) Depressive symptoms	4.88 (3.71); 0–17	0.31^∗∗^	0.73^∗∗^	–					
(4) Parenting stress	17.54 (2.99); 3–21	0.26^∗∗^	0.47^∗∗^	0.48^∗∗^	–				
**Mindful parenting**									
(5) Listening with full attention	18.59 (2.91); 9–25	-0.34^∗∗^	-0.40^∗∗^	-0.40^∗∗^	-0.44^∗∗^	–			
(6) Emotional awareness of the child	11.70 (1.74); 7–15	-0.18^∗∗^	-0.21^∗∗^	-0.24^∗∗^	-0.33^∗∗^	0.39^∗∗^	–		
(7) Self-regulation in parenting	27.05 (4.14); 14–40	-0.24^∗∗^	-0.44^∗∗^	-0.45^∗∗^	-0.51^∗∗^	0.53^∗∗^	0.38^∗∗^	–	
(8) Non-judgmental acceptance of	24.30 (4.21); 13–35	-0.21^∗∗^	-0.55^∗∗^	-0.47^∗∗^	-0.41^∗∗^	0.37^∗∗^	0.26^∗∗^	0.49^∗∗^	–
parental functioning
(9) Compassion for the child	25.77 (2.99); 17–30	-0.13^∗^	-0.27^∗∗^	-0.27^∗∗^	-0.43^∗∗^	0.50^∗∗^	0.48^∗∗^	0.57^∗∗^	0.34^∗∗^

^∗^*p < 0.05, ^∗∗^*p* < 0.01*.

### Comparison Analyses as a Function of Working Variables

Differences in study variables as a function of work schedule (fixed versus flexible versus shift work), type of employment (full-time versus part-time/occasional), and number of working hours per week (less than 40 versus 40 or more) are presented in Table 3. Significant differences in work-family conflict were found for work schedule, type of employment and number of weekly working hours. Specifically, parents with a shift work schedule presented significantly higher levels of work-family conflict than those with a fixed or flexible schedule (no differences were found between fixed and flexible schedules), and parents working full-time and 40 h or more reported higher levels of work-family conflict than those working part-time and less than 40 h per week, respectively. Significant differences in mindful parenting dimensions were found only for work schedule [Wilk’s Lambda = 0.921, *F*(10,656) = 2.74, *p* = 0.003]. The examination of the univariate effects revealed that differences were only significant for the self-regulation in parenting and non-judgmental acceptance of parental functioning dimensions, with parents with a flexible schedule reporting significantly higher levels of self-regulation and of non-judgmental acceptance than parents with a shift work schedule (no significant differences were found between fixed and flexible or shift work schedules).

**Table 3:** Comparison analyses as a function of working variables.

	Work schedule *M (SD)*				Type of employment *M (SD)*			Weekly working hours *M (SD)*		
	Fixed	Shift	Flexible		Full-time	Part-time/		<40 h	≥40 h
	*n* = 234	*n* = 54	*n* = 47	Comparison analyses	*n* = 296	occasional *n* = 39	Comparison analyses	*n* = 141	*n* = 194	Comparison analyses
Work-family conflict	18.50 (7.35)	23.78 (7.25)	17.72 (6.47)	*F*(2,332) = 12.96, ***p* < 0.001**	19.74 (7.24)	15.41 (8.15)	*F*(1,333) = 11.98, ***p* < 0.001**	17.55 (7.48)	20.47 (7.24)	*F*(1,332) = 12.95, ***p* < 0.001**
Anxiety	6.71 (3.68)	6.78 (4.39)	6.23 (3.94)	*F*(2,332) = 0.13, *p* = 0.978	6.64 (3.78)	7.00 (4.14)	*F*(1,333) = 0.30, *p* = 0.583	6.87 (3.87)	6.55 (3.79)	*F*(1,332) = 0.59, *p* = 0.442
Depression	4.81 (3.63)	5.52 (3.97)	4.51 (3.81)	*F*(2,332) = 1.08, *p* = 0.342	4.81 (3.60)	5.44 (4.48)	*F*(1,333) = 0.99, *p* = 0.321	5.12 (3.88)	4.71 (3.58)	*F*(1,332) = 1.02, *p* = 0.314
Parenting stress	39.18 (6.45)	37.98 (6.33)	37.66 (5.35)	*F*(2,332) = 1.66, *p* = 0.191	38.83 (6.24)	38.38 (6.30)	*F*(1,333) = 0.17, *p* = 0.681	38.83 (6.74)	38.73 (5.99)	*F*(1,332) = 0.18, *p* = 0.895
MP: Listening with full attention	18.74 (3.01)	17.93 (3.00)	18.62 (2.19)	*F*(2,332) = 1.72, *p* = 0.181	18.61 (2.96)	18.46 (2.57)	*F*(1,333) = 0.09, *p* = 0.768	18.55 (2.87)	18.63 (2.95)	*F*(1,333) = 0.06, *p* = 0.810
MP: Emotional awareness of the child	11.76 (1.70)	11.78 (1.84)	11.32 (1.78)	*F*(2,332) = 1.35, *p* = 0.260	11.66 (1.74)	12.03 (1.72)	*F*(1,333) = 1.51, *p* = 0.219	11.74 (1.74)	11.68 (1.73)	*F*(1,333) = 0.13, *p* = 0.718
MP: Self-regulation in parenting	27.09 (4.26)	25.83 (3.76)	28.23 (3.56)	*F*(2,332) = 4.36, ***p* = 0.014**	27.00 (4.09)	27.41 (4.49)	*F*(1,333) = 0.34, *p* = 0.561	26.82 (4.28)	27.22 (4.04)	*F*(1,333) = 0.77, *p* = 0.382
MP: Non-judgmental acceptance of parental functioning	24.18 (4.19)	23.63 (4.24)	25.66 (4.07)	*F*(2,332) = 3.29, ***p* = 0.039**	24.27 (4.20)	24.46 (4.33)	*F*(1,333) = 0.07, *p* = 0.794	24.13 (3.93)	24.42 (4.41)	*F*(1,333) = 0.39, *p* = 0.534
MP: Compassion for the child	25.67 (3.03)	25.93 (3.08)	26.06 (2.70)	*F*(2,332) = 0.43, *p* = 0.653	25.73 (2.98)	26.03 (3.13)	*F*(1,333) = 0.33, *p* = 0.567	25.77 (2.90)	25.76 (3.07)	*F*(1,333) = 0.00, *p* = 0.976

*MP, mindful parenting. Significant differences are marked as bold*.

### Comparison Analyses as a Function of Parents’ Gender

Differences in study variables as a function of parents’ gender are presented in Table 4. The only significant difference was found for listening with full attention, with fathers presenting higher levels of this mindful parenting dimension than mothers.

**Table 4:** Comparison analyses as a function of parents’ gender.

	Mothers	Fathers
	(*n* = 289)	(*n* = 49)
	*M* (*SD*)	*M* (*SD*)	Comparison analyses
Work-family conflict	18.50 (7.35)	17.72 (6.47)	*F*(1,333) = 1.91, *p* = 0.168
Anxiety	6.75 (3.85)	6.24 (3.66)	*F*(1,333) = 0.72, *p* = 0.397
Depression	4.93 (3.77)	4.59 (3.38)	*F*(1,333) = 0.33, *p* = 0.564
Parenting stress	38.81 (6.48)	38.57 (5.11)	*F*(1,333) = 0.06, *p* = 0.807
**Mindful parenting**			
Listening with full attention	18.46 (2.98)	19.43 (2.34)	*F*(1,333) = 4.52, *p* = 0.034
Emotional awareness of the child	11.75 (1.72)	11.39 (1.83)	*F*(1,333) = 1.74, *p* = 0.188
Self-regulation in parenting	27.54 (3.46)	26.97 (4.23)	*F*(1,333) = 0.77, *p* = 0.382
Non-judgmental acceptance of parental functioning	24.20 (4.17)	24.91 (4.42)	*F*(1,333) = 1.15, *p* = 0.285
Compassion for the child	25.73 (3.05)	25.98 (2.64)	*F*(1,333) = 0.27, *p* = 0.607

### The Indirect Effect of Work-Family Conflict on Mindful Parenting Through Parental Anxiety/Depressive Symptoms and Parenting Stress

**Figure 1:**
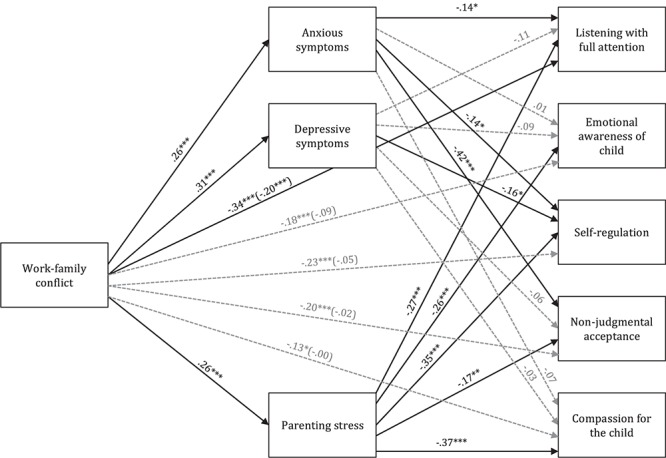
Path model examining the associations between work-family conflict and mindful parenting dimensions through anxiety, depression, and parenting stress. Path values represent standardized regression coefficients. For simplicity, measurement error terms and covariates are not shown. Gray dashed arrows represent the non-significant associations between variables. Direct effects are shown inside parentheses and total effects are shown outside parentheses. ^∗^*p* < 0.05, ^∗∗^*p* < 0.01, ^∗∗∗^*p* < 0.001.

A path model was tested to explore the indirect effects of work-family conflict on mindful parenting dimensions. Sociodemographic and working variables that were significantly correlated with mindful parenting dimensions were introduced as covariates in the model. The initial path model presented a poor fit to the data, χ^2^(60) = 641.59, *p* < 0.001; CFI = 0.500; SRMR = 0.156; RMSEA = 0.170, *p* < 0.001, 90% CI = [0.16, 0.18]. Therefore, we examined the modification indices, which suggested that some residuals might be correlated. We initially allowed the correlation between the residuals belonging to depression, anxiety and parenting stress and between income and education, which presented the largest modification indices. Although the model fit improved significantly [Δχ^2^(4) = 349.13, *p* < 0.001], it did not achieve a good fit (χ^2^(56) = 292.46, *p* < 0.001; CFI = 0.797; SRMR = 0.089; RMSEA = 0.112, *p* < 0.001, 90% CI = [0.10, 0.13]). Therefore, some residuals from mindful parenting dimensions were also allowed to correlate (listening with full attention with emotional awareness, self-regulation, and compassion for the child; emotional awareness with self-regulation and compassion for the child; self-regulation with non-judgmental acceptance and compassion for the child). The respecified path model presented a very good fit to the data, χ^2^(49) = 83.46, *p* = 0.002; CFI = 0.970; SRMR = 0.060; RMSEA = 0.046, *p* = 0.638, 90% CI = [0.03, 0.06] (see Figure 1).

As presented in Table 5, work-family conflict had an indirect effect on all mindful parenting dimensions through depressive and anxiety symptoms and parenting stress. When analyzing the specific indirect effects, we found that work-family conflict was associated with listening with full attention through anxiety and parenting stress; with emotional awareness of the child through parenting stress; with self-regulation through anxiety, depression and parenting stress; with non-judgmental acceptance through anxiety and parenting stress; and with compassion for the child only through parenting stress.

**Table 5:** Indirect effects of work-family conflict on mindful parenting dimensions.

	Estimate	*p*-value	BC 95%CI
			Lower/Upper
**Indirect effects**			
WFC → LFA	-0.140	0.001	-0.205/-0.082
WFC → EAC	-0.096	0.001	-0.151/-0.048
WFC → SR	-0.179	0.001	-0.253/-0.114
WFC → NJAPF	-0.173	0.001	-0.246/-0.109
WFC → CC	-0.127	0.001	-0.198/-0.069
**Specific indirect effects**			
WFC → Depression → LFA	-0.014	0.127	-0.036/0.003
WFC → Depression → EAC	-0.007	0.287	-0.022/0.006
WFC → Depression → SR	-0.027	0.012	-0.057/-0.006
WFC → Depression → NJAPF	-0.011	0.382	-0.037/0.015
WFC → Depression → CC	-0.004	0.661	-0.027/0.016
WFC → Anxiety → LFA	-0.014	0.038	-0.036/-0.001
WFC → Anxiety → EAC	0.001	0.940	-0.010/0.010
WFC → Anxiety → SR	-0.021	0.030	-0.047/-0.002
WFC → Anxiety → NJAPF	-0.062	0.001	-0.095/-0.034
WFC → Anxiety → CC	-0.008	0.290	-0.027/0.007
WFC → Parenting Stress → LFA	-0.027	0.001	-0.045/-0.013
WFC → Parenting Stress → EAC	-0.016	0.001	-0.027/-0.008
WFC → Parenting Stress → SR	-0.050	0.001	-0.077/-0.028
WFC → Parenting Stress → NJAPF	-0.024	0.002	-0.044/-0.009
WFC → Parenting Stress → CC	-0.039	0.001	-0.060/-0.021

*WFC, work-family conflict; LFA, listening with full attention; EAC, emotional awareness of the child; SR, self-regulation in parenting; NJAPF, non-judgmental acceptance of parental functioning; CC, compassion for the child. Standardized coefficients are presented for the global indirect effects and unstandardized coefficients are presented for the specific indirect effects*.

### Structural Invariance Across Children’s Ages and Parents’ Gender

Multigroup analyses were performed to test the structural invariance of the path model across children’s age groups (toddlers versus preschool and grade school children versus adolescents) and parents’ gender. Concerning the invariance analyses for children’s age groups, the baseline model demonstrated a good fit to the data in the group of parents of preschool and grade school children, χ^2^(49) = 69.98, *p* = 0.016; CFI = 0.971; SRMR = 0.068; RMSEA = 0.046, *p* = 0.581; 90% CI = [0.02, 0.07]. Among parents of toddlers and adolescents, the model presented a poorer fit to the data (toddlers: χ^2^(49) = 64.06, *p* = 0.073; CFI = 0.948; SRMR = 0.100; RMSEA = 0.065, *p* = 0.279; 90% CI = [0.00, 0.11]; adolescents: χ^2^(49) = 73.28, *p* = 0.014; CFI = 0.865; SRMR = 0.112; RMSEA = 0.092, *p* = 0.074; 90% CI = [0.04, 0.13]). Then, we compared the unconstrained and constrained models (unconstrained: χ^2^(147) = 207.92, *p* < 0.001; CFI = 0.949; SRMR = 0.101; RMSEA = 0.035, *p* = 0.990; 90% CI = [0.02, 0.05]; constrained: χ^2^(209) = 269.11, *p* = 0.003; CFI = 0.950; SRMR = 0.107; RMSEA = 0.029, *p* = 1.00; 90% CI = [0.02, 0.04]) and found that the difference between both models was not significant, Δχ^2^(62) = 61.19, *p* = 0.505, which indicates that the relationships between the variables in the model were invariant across groups.

To explore if the path model was invariant across mothers and fathers, another multigroup analysis was performed. In this analysis, parents’ gender was not introduced as a covariate. The baseline model for mothers demonstrated a good fit to the data (χ^2^(37) = 51.47, *p* = 0.057; CFI = 0.986; SRMR = 0.058; RMSEA = 0.037, *p* = 0.815; 90% CI = [0.00, 0.06]); however, the model did not present a good fit in the group of fathers (χ^2^(37) = 67.59, *p* = 0.002; CFI = 0.815; SRMR = 0.131; RMSEA = 0.136, *p* = 0.008; 90% CI = [0.08, 0.19]). The comparison between the unconstrained and the constrained models (unconstrained: χ^2^(74) = 120.02, *p* < 0.001; CFI = 0.961; SRMR = 0.131; RMSEA = 0.043, *p* = 0.779, 90% CI = [0.03, 0.06]; constrained: χ^2^(104) = 146.86, *p* = 0.004; CFI = 0.964; SRMR = 0.143; RMSEA = 0.035, *p* = 0.975, 90% CI = [0.02, 0.05]) revealed a non-significant difference, Δχ^2^(30) = 26.84, *p* = 0.632, which indicates that the relationships between variables in the model were invariant across groups.

## Discussion

This study had two main goals. First, we aimed to investigate whether some important work-related variables, such as type of employment, work schedule and number of working hours per week, could play a role in parents’ work-family conflict and also on their emotional distress (anxiety/depression symptoms, parenting stress) and parenting practices (i.e., mindful parenting). We found that parents with a shift work schedule, parents with a full-time job, and parents who work 40 h or more per week had higher levels of work-family conflict than parents with a flexible or fixed work schedule, parents with a part-time job and parents who work less than 40 h per week, respectively. We also found that parents with a flexible work schedule had higher levels of self-regulation in parenting and higher levels of non-judgmental acceptance of parental functioning than parents with a fixed or shift work schedule. Another major goal of this study was to investigate whether work-family conflict was linked to mindful parenting dimensions through parental psychopathology and parenting stress. Overall, we found that higher levels of work-family conflict were indirectly associated with lower levels of mindful parenting dimensions through anxiety and depression symptoms and parenting stress. This model was shown to be invariant between mothers and fathers and between parents of children from different age groups. These results will be discussed in further detail below.

Corroborating the results of previous studies (e.g., McLoyd et al., 2008; Cho, 2018), we found that parents working full-time and 40 h or more per week and with a shiftwork schedule reported higher levels of work-family conflict. Parents working full-time and more than 40 h per week have objectively less time for their family, which can lead them to experience greater difficulty in successfully managing family and work roles. Parents working fewer hours and part-time have more time and, consequently, more opportunities to be with their family, which seems to be a key factor for experiencing lower levels of work-family conflict. In addition, parents with a shift work schedule usually work on weekends, work evening and night shifts, and have irregular or rotating shifts. This type of work schedule, often called “unsociable work” (Strazdins et al., 2006), may be highly disruptive for family routines (McLoyd et al., 2008) and may lead parents to experience a greater instability and a lower perception of control over their lives and to have less time and energy resources for performing their family role, which may result in a greater perception that work demands are negatively interfering with their family demands (Cho, 2018).

The results of our study also suggest that flexible work schedules can be protective of parenting. These findings are in line with those of previous investigations that found that working time flexibility can facilitate the work-life balance of employees and, particularly, of parents (Riedmann et al., 2006; OECD, 2011; Eurofound, 2016). Interestingly, in a study that included a large sample of working parents of children aged 0–16 from the United Kingdom (Bowden, 2009), 51% of the working parents reported that they would have a better relationship with their children if they had a flexible work schedule; and 63% of the parents working full-time reported that their current work schedule prevent them from being with their children the amount of time they would like. However, to the best of our knowledge, our study is the first study directly exploring the role of flexible working hours on parenting behaviors. Specifically, we found that parents with flexible schedules presented higher levels of self-regulation in parenting and of non-judgmental acceptance of parental functioning than parents with fixed or shiftwork schedules. Having flexible working hours allows parents to better reconcile work demands and tasks with their family role (e.g., parents can change their workday start and finish times in order to do some activity with their children or work from home if their child is sick) (OECD, 2016). This greater balance may lead parents to feel less stressed and better able to regulate their emotions and behaviors when interacting with their children (e.g., as suggested by their higher levels of self-regulation in parenting, they may have a greater ability to pause before reacting to a child’s negative behaviors, instead of automatically displaying hostility or negative affect), which in turn may have a positive impact on the relationship with their children. In addition, being able to manage working hours allows parents to avoid missing important moments in their children’s lives (e.g., school parties and sport events), to be present when necessary (e.g., when children are sick), and to involve themselves in a greater number of leisure activities with their children. This can make parents less likely to feel that they have failed as parents, which can lead them to have higher levels of non-judgmental acceptance of their parental functioning. Parents who work in shifts or who have a fixed work schedule are less able to manage their time according to their child’s needs (Barnett et al., 2008), which may lead them to feel guilty for not being present whenever necessary and to criticize themselves as parents.

We also analyzed gender differences in study variables. No differences were found in any variable, with the exception of the mindful parenting dimension listening with full attention. While it is important to keep in mind that our study comprised a very small number of fathers (13.7% of the total sample) and that our group of fathers was a self-selected sample (most fathers were recruited online; therefore, these fathers are likely those who are more involved in childcare and who are more interested in parenting-related issues), our results suggest that the mothers and fathers in our sample experience similar levels of work-family conflict, anxiety/depression symptomatology, and parenting stress. In the majority of contemporary families, both parents work outside the home and both take an active role in caring for their children. In fact, according to the OECD data on gender equality, although Portuguese women are less likely to be employed than men, the gender gap is smaller than the OECD mean (10.8% points). Specifically, in 2017, in Portugal, the female employment rate (64.8%) was only 6.3% points lower than the average employment rate for men (71.1%) (OECD, 2019). However, even though men and women are expected to take equal roles in the family and working contexts, the traditional gender-role stereotypes are still prevalent, particularly in dual-earner families with children (Endendijk et al., 2018). While women are expected to succeed at work, they are also expected to assume the most prominent role in childcare, and while men are still expected to be the main economic providers, many also want to have an active role in their families (which may particularly be the case for the fathers who constituted our sample). Thus, it is not surprising that both report similar levels of work-family conflict and emotional distress.

With regard to gender differences in mindful parenting, it is interesting to note that in our sample, fathers reported higher levels of listening with full attention than mothers, a dimension that refers to the parents’ ability to direct their attention and awareness to the child and be fully present during parent–child interactions. A possible explanation is that mothers, because they still assume the most salient roles in the childcare and at home and spend more time than fathers with their children (Laflamme et al., 2002), may feel a greater difficulty in balancing all the tasks and roles as mothers, homemakers, workers, among others, which may have a negative impact on their ability to be in the present moment with their children. Although they seem to not perceive a higher level of work-family conflict than fathers, they may be more concerned with endless outstanding tasks (in the different contexts in which they play a role), and these constant concerns and role demands may deprive them of the ability to direct their full attention to their children when interacting with them. A typical example would be a mother who is thinking about what she is going to do for dinner and about the chores left to do at her job while playing with her child. In fact, extensive research shows that fathers tend to spend a greater amount of their interaction time with their children in play activities, whereas mothers usually spend more time in caregiving activities (McBride and Mills, 1993; Tiedje and Darling-Fisher, 1993). Play activities are likely to involve more attention to the present moment than caregiving activities, such as feeding the child or helping to do homework, which can also explain our results. Lastly, women are known to be more ruminative than men (Johnson and Whisman, 2013), which can also explain our results. Whereas men can more easily turn off their preoccupations and enjoy the present moment while interacting with their children, women may more easily engage in ruminative thoughts that divert their attention from what is going on in the present moment when they are with their children.

With regard to the path model, we found that, although work-family conflict was indirectly associated with all mindful parenting dimensions through anxiety, depression, and/or parenting stress, it only had a direct effect on the listening with full attention dimension. This direct effect suggests that perceiving a greater level of interference of work with family life (particularly with regard to less quality time with the family and increased irritability at home, which are the main aspects assessed by the work-family conflict measure we used in this study) can translate into a diminished ability to listen to the child and engage in activities together in a calm and attentive way. Objectively, those who have fewer opportunities to be with their family because of work also have fewer opportunities to bring their mindful awareness to interactions with their children. In addition, we may hypothesize that parents with higher levels of work-family conflict may be absorbed by work-related concerns or by ruminations related to the frustration they feel at having so little time for the family, which may consume their attention resources and leave them with a diminished ability to concentrate on the present when they are with their children. For example, if parents are worried about a deadline, an outstanding task, or any other work-related issue, or if parents are focused on the fact that they usually have little time to be with their children (“I should have more time for my family”), they may be so consumed by those thoughts and concerns that they may not be able to be fully present and focus entirely on what is going on when they are interacting with their children (e.g., playing, feeding them).

As mentioned, we found several indirect effects between work-family conflict and mindful parenting through anxiety, depression, and/or parenting stress. These indirect effects occurred through positive associations between work-family conflict and the three mediators and negative associations between the mediators and the mindful parenting dimensions. With regard to the positive associations between work-family conflict and anxiety, depression, and parenting stress, these results are in accordance with previous investigations that have shown that work-family conflict is an important risk factor for parental psychopathology and parenting stress (e.g., Vieira et al., 2012; Westrupp et al., 2016). Managing family and work roles and feeling that the demands of time and energy these roles require are not easily reconcilable can be profoundly exhausting and distressing for parents and make them feel anxious, depressed, and highly stressful in their parental roles. Parents may feel that to respond satisfactorily to the demands of their jobs, they fail as parents, which may undermine their psychological well-being and lead them to feel that the demands of being a parent exceed their personal and social resources (e.g., parenting skills, social support) to cope with those demands (Abidin, 1992).

With regard to the links between the mediators (anxiety, depression, and parenting stress) and the mindful parenting dimensions, we found that while parenting stress was negatively associated with all mindful parenting dimensions (i.e., dimensions related to the parents, the child, and the parenting relationship), anxiety and depression were predominantly associated with parent-centered dimensions. Specifically, anxiety was negatively associated with self-regulation, non-judgmental acceptance of the parenting functioning and listening with full attention, and depression was only negatively associated with self-regulation. These findings suggest that individual factors, such as psychopathology, seem to play a more relevant role on mindful parental behaviors that are more focused on the parent themselves (the ability to listen with full attention, to regulate emotions and behaviors in parent–child interactions and to non-judgmentally accept parental mistakes and limitations), whereas parenting stress seem to have a broader effect and to also play a role in dimensions more focused on the child, such as the emotional awareness of child and the compassion for the child.

With regard to parenting stress, several previous studies have already shown a consistent association between this type of stress and mindful parenting (e.g., Bögels et al., 2014; Gouveia et al., 2016; Moreira and Canavarro, 2018b). While previous research has typically explored this link in the opposite direction to that we investigated in this study, suggesting that mindful parenting may create favorable conditions for parents to experience lower levels of parenting stress, the opposite direction is also valid. Several theories of parenting stress argue that this type of stress leads to dysfunctional parenting (Abidin, 1992; Deater-Deckard, 2004), which is consistent with the results of our study that suggest that parents who are more stressed due to the demands and challenges of their parenting roles may struggle to adopt a mindful stance in parenting. Perceiving parental demands (e.g., survival demands such as feeding and protection and psychological demands such as giving affection to the child and helping the child regulate emotions) as exceeding parental resources (e.g., feelings of competence, time, instrumental, and emotional support) may lead parents to experience parenting as more stressful and taxing than rewarding, which is not the ideal condition for mindful parenting. This study adds to the existing knowledge by showing that experiencing work-related stress can spill over to the parenting context, leading parents to experience higher levels of parenting stress and, in turn, less mindful parenting.

Our model also suggests that parents’ anxiety and depression symptoms also play a role, albeit a less prominent role than parenting stress, in explaining why work-family conflict is associated with lower levels of mindful parenting. Several specific indirect effects were found, which corroborate the large body of research indicating that parental psychopathology suffers the influence of work-related variables (Westrupp et al., 2016) and is one of the most influential determinants of maladaptive parenting practices (Belsky, 1984; Harvey et al., 2011). We found that both depression and anxiety were associated with lower levels of self-regulation in parenting. Anxious and depressed individuals tend to have greater difficulties in regulating their own emotions (Gross and Jazaieri, 2014), and this difficulty may extend to the parent–child relationship. In fact, our results suggest that more anxious and/or depressed parents may have greater difficulty in regulating their emotions and behaviors when interacting with their children and a greater predisposition to be more impulsive and reactive to children’s (negative) behaviors. These results are consistent with previous studies showing, for instance, that parental depression increases parents’ child-directed hostility and negativity (Lovejoy et al., 2000; Harvey et al., 2011), which often is the result of a lower ability to regulate their own negative emotions.

In addition, we found that higher levels of anxiety were a mediator of the relationship between work-family conflict and listening with full attention. This result corroborate previous studies showing, for instance, that anxious parents tend to be less engaged and more withdrawn during interactions with their children (Woodruff-Borden et al., 2002), to have lower levels of sensitivity when interacting with their children (Nicol-Harper et al., 2007), and to be less warm (Whaley et al., 1999; Williams et al., 2012) than non-anxious parents. Several explanations may underlie our results. First, anxious parents may focus their attention primarily on themselves and on their own needs, as suggested by previous studies that have shown that parental psychopathology promotes self-focused attention, particularly on their own symptoms and needs (Ingram, 1990; Dix and Meunier, 2009). By focusing their attention on themselves and on their own symptoms of anxiety, anxious parents are less likely to direct their attention to their child, as suggested by the negative link between anxiety and listening with full attention. Second, anxious mothers might be more ruminative and, consequently, less able to be mindful in the interactions with their children. This hypothesis is consistent with previous research showing that parental rumination has a negative effect on parent–child interactions and relationship outcomes (e.g., Stein et al., 2012; O’Mahen et al., 2015), as well as with previous studies showing that rumination and mindfulness are negatively associated (e.g., Teasdale et al., 1995; Schut and Boelen, 2017). Rumination, which is highly prevalent among individuals with anxiety disorders (Dar and Iqbal, 2015), may monopolize attentional and cognitive resources and narrow parents’ attentional focus, thereby making it difficult for mothers to fully engage with their children and to be fully present when interacting with them (Stein et al., 2012; Moreira and Canavarro, 2018b). Since we found that work-family conflict was linked to anxiety and, consequently, to the ability to listen to the child with full attention, it can be hypothesized that these ruminative thoughts are linked to work-related features. Future studies should further investigate this possibility by using a measure that evaluates rumination that is particularly focused on work-related issues.

Anxiety symptoms also mediated the link between work-family conflict and non-judgmental acceptance of parental functioning. These associations are consistent with previous research that has shown that distressed mothers are usually more self-critical and, consequently, are more likely to endorse a negative view of themselves as mothers (Goodman and Gotlib, 1999). Self-criticism is a transdiagnostic factor that is linked to several forms of psychopathology (Blatt and Zuroff, 1992;Gilbert and Procter, 2006). Therefore, it is likely that more anxious parents (as well as parents showing higher levels of parenting stress) might find it difficult to accept perceived limitations as parents and feel that they do not meet their self-defined standards in the relationship with their children, particularly if they experience a higher level of work-family conflict, as suggested by the results of this study.

The invariance analyses revealed that the relationships in the model were independent of the parents’ gender and of the child’s developmental stage. With regard to parents’ gender, and although men and women struggle with different gender-role stereotypes, work-family conflict seems to play an identical role in mothers’ and fathers’ emotional distress and in their parenting practices. Given the large number of dual-earner families in today’s society, it is possible that, in some cases, both parents may have high levels of work-family conflict, which may have a cumulative effect on the risk of less mindful parenting practices. Furthermore, although the developmental challenges are different in young children, school-aged children and adolescents (e.g., Teasdale et al., 1995; Schut and Boelen, 2017), the effect of work-family conflict on parental distress and, in turn, on mindful parenting seems to be transversal to the different developmental stages. However, it is important to note that the baseline model for the parents of toddlers and adolescents and for fathers did not present a very good fit to the data. While this may mean that the path model does not fit these groups well, these results may also be a consequence of the reduced size of these subsamples. Future studies should seek to include the same number of parents of children of different ages and the same number of mothers and fathers.

### Limitations of the Study

This study presents several limitations that should be noted. First, this study has a cross-sectional design, which does not allow for determining with confidence the direction of the associations between the variables. For instance, it is also possible that experiencing higher levels of psychopathology symptoms and parenting stress leads to a greater strain between work and family demands. On the other hand, mindful parenting can also predict parenting stress and even work-family conflict. For instance, more mindful parents may be more able to positively manage the challenges and demands of parenting, consequently feeling less parenting stress. Mindful parents might also manage in a more balanced way the demands of work and family, feeling less conflict between these two roles. Future longitudinal studies should be conducted to understand the direction of the associations between these variables. In addition, and although we have considered psychopathology symptoms and parenting stress as parallel mediators, future longitudinal studies, in which all variables are assessed through several time points, may contribute to clarify the directionally between these variables. Second, 86.3% of the participants were mothers, which limits the generalizability of the results to fathers. Future studies should include a larger number of fathers to explore in further detail gender effects on the associations studied. Third, most parents were married or living with a partner, had completed higher education, had a child between the ages of 3 and 10 years old, and had a full-time job and a fixed work schedule, which may limit the generalization of results to parents with other sociodemographic characteristics, with younger or older children (i.e., toddlers and adolescents) and with other work conditions. Fourth, the sample was recruited online and at schools. Although no differences were found in study variables as a function of the local of recruitment, the two groups differed in some sociodemographic variables. In addition, online recruitment is often associated with a self-selection bias (i.e., parents who participate in an online study tend to be more interested in the study theme and to be more motivated to complete the questionnaires), which may compromise the representativeness of the sample. Fifth, the Cronbach’s alpha of the Emotional Awareness of the Child subscale was below the recommended threshold of 0.70. Although some authors argue that Cronbach’s alphas of 0.60 are acceptable in research in the social sciences (Galinsky, 1987), some caution should be used when interpreting the results obtained with this subscale. Finally, to improve the model fit of the path model, we have allowed some residuals of the study variables to correlate. However, this was made not only based in statistical criteria but also based on a theoretical rationale (e.g., it is expectable that the associations between anxiety and depression symptoms were high, so we allowed the residuals to be correlated, which suggests that other factors not included in the model may influence both anxiety and depression symptoms).

### Practical Implications of the Study

The results of the present study provide further evidence that certain working conditions (e.g., shiftwork) and work-family conflict may have an adverse impact on the lives of employed parents, particularly on their mental health and parental behaviors. It is critically important that policy makers and employers recognize this impact and devise strategies that can help parents better balance their work and family responsibilities. With the growing rates of dual-earner families, it is increasingly important that workplaces adopt and implement family-friendly policies that can minimize the impact of work on family, such as more extended maternity and paternity leaves (in Portugal, the maternity leave has a maximum of 6 months and the paternity leave is 25 days), sickness leaves, career breaks and extended leave, part-time work, and flexible work arrangements (e.g., alternate work schedule, teleworking, compressed work week) (Aron et al., 2013; Hair et al., 2013). The results of our study emphasize the protective role of a flexible work schedule and the detrimental role of a shiftwork schedule on parent’s wellbeing and parenting. Family-friendly policies can help working parents have a more balanced life, which may benefit not only the parents themselves but also their children, their families, and even their organizations. For instance, several studies have demonstrated that family-friendly policies promote a greater commitment to work and higher levels of job satisfaction (Yu, 2018), greater productivity (Dex and Smith, 2002; Bae and Yang, 2017) and fewer turnover intentions (Bae and Yang, 2017).

In addition, our study suggests that work-family conflict plays a detrimental role on parents’ mental health and parenting practices at different stages of child development, particularly during preschool and grade school years. Although most family support policies in Portugal are designed for parents in the postpartum period (e.g., maternity leave and breastfeeding breaks), our study draws attention to the importance of supporting parents in the later stages of child development. Therefore, family-friendly work arrangements should include parents of children and adolescents of all ages and should not be exclusive of parents during the postpartum period.

When it is not possible to change work conditions (e.g., to change from a shiftwork schedule to a flexible schedule) and/or when workplaces are not willing to implement family-friendly policies, targeted interventions for parents who are experiencing high levels of stress and difficulty in reconciling work and family demands, aimed at helping them develop strategies to better cope with the work-family conflict, can be very useful. In addition, preventive parenting interventions designed to promote more positive and mindful parenting practices, regardless of parents’ work conditions, should consider the impact that work can have on parents’ mental health and parenting and, therefore, help them to develop skills that allow them to more adaptively balance work and family responsibilities (e.g., time management skills) in order to prevent them from experiencing high levels of conflict between their work and family roles.

Another result of our study with important implications is that anxiety, rather than depression, seems to play an important role in how parents see themselves as parents and in their ability to be fully present when interacting with their children. These findings underline the importance of assessing, in clinical context, the relationship that anxious parents establish with their children, in order to provide them strategies that not only reduce their anxiety but also promote a greater acceptance of perceived mistakes and limitations as parents, a greater self-regulation in parenting, and a greater ability to defuse from their problems and to be present when interacting with their children. Although the literature has predominantly emphasized the negative role that parental depression plays in parenting behaviors and in child development (Yu, 2018), our study draws attention to the important role of anxious symptomatology. In addition, parenting stress has been shown to be the mediator that best explains the relationship between work-family conflict and mindful parenting, since it has been shown to be associated with all the dimensions of this parenting style. It seems to be parenting stress that has a greater impact on mindful parenting behaviors, particularly on child-centered dimensions (which were not associated with psychopathology). These results underscore the importance and utility of mindfulness focused parental interventions for parents with high levels of parenting stress (Lovejoy et al., 2000), particular for those whose the likely cause of stress is a conflict between work and family demands. These results also point out that a more general preventive approach targeting the promotion of mental health in work contexts may expand its benefits not only at the individual level, but also to the parent–child relationship.

Promoting mindful parenting, namely, by creating better working conditions so that parents can effectively *be* mindful in the relationship with their children, is extraordinarily important not only for parents but also for children. Mindful parenting can be a privileged vehicle to foster a positive and secure relationship between the parents and the children (Bögels and Restifo, 2014), and in several studies, it has been shown to promote better psychosocial adjustment in various groups of children and adolescents (e.g., less internalizing and externalizing behaviors, greater wellbeing) (Medeiros et al., 2016; Moreira et al., 2018b). Therefore, the promotion and implementation of work policies that protect the family, by reducing work-family conflict and thus preventing the development of psychopathology and parental stress and promoting a mindful stance in parenting, are fundamental for future generations. Parents who are happier in their different work and family roles are also parents who are more balanced and mindful in the relationship with their children.

## Data Availability

The datasets generated for this study are available on request to the corresponding author.

## Ethics Statement

All procedures performed in studies involving human participants were in accordance with the ethical standards of the institutional and/or national research committee and with the 1964 Helsinki declaration and its later amendments or comparable ethical standards. The Ethics Committee of the Faculty of Psychology and Education Sciences of the University of Coimbra approved the study. Informed consent was obtained from all participants included in the study. Participants recruited through a data collection website provided informed consent by clicking on the option “I understood and accept the conditions of the study.” Participants recruited at the school provided written informed consent.

## Author Contributions

HM designed the study, performed the data analyses, and wrote the manuscript. AF designed the study and revised the final draft of the manuscript. BC contributed to the interpretation of data. MC collaborated in the editing of the final manuscript.

## Conflict of Interest Statement

The authors declare that the research was conducted in the absence of any commercial or financial relationships that could be construed as a potential conflict of interest.
